# Correlation of microbiological yield with radiographic activity on chest computed tomography in cases of suspected pulmonary tuberculosis

**DOI:** 10.1371/journal.pone.0201748

**Published:** 2018-08-09

**Authors:** Yousang Ko, Ho Young Lee, Yong Bum Park, Su Jin Hong, Jeong Hwan Shin, Seok Jin Choi, Changhwan Kim, So Young Park, Jin Young Jeong

**Affiliations:** 1 Division of Pulmonary, Allergy and Critical Care Medicine, Department of Internal Medicine, Kangdong Sacred Heart Hospital, Hallym University College of Medicine, Seoul, Republic of Korea; 2 Lung Research Institute of Hallym University College of Medicine, Chuncheon, Republic of Korea; 3 Division of Pulmonary, Allergy and Critical Care Medicine, Department of Internal Medicine, Busan Paik Hospital, Inje University College of Medicine, Busan, Republic of Korea; 4 Department of Radiology, Hanyang University Guri Hospital, Hanyang University College of Medicine, Guri, Republic of Korea; 5 Department Laboratory Medicine, Busan Paik Hospital, Inje University College of Medicine, Busan, Republic of Korea; 6 Department of Radiology, Busan Paik Hospital, Inje University College of Medicine, Busan, Republic of Korea; 7 Department of Internal Medicine, Jeju National University Hospital, Jeju, Republic of Korea; 8 Department of Pulmonary and Critical Care Medicine, Chung Nam National University Medical Center, Daejeon, Republic of Korea; 9 Hallym Research Institute of Clinical Epidemiology, Chuncheon, Republic of Korea; Fundació Institut d’Investigació en Ciències de la Salut Germans Trias i Pujol, Universitat Autònoma de Barcelona, SPAIN

## Abstract

**Background:**

Little is known about the correlation between microbiological yield and radiographic activity, on chest computed tomography (CT), in suspected pulmonary tuberculosis (PTB) cases, despite CT being widely used, clinically.

**Methods:**

We used multicenter retrospective data, obtained from medical records, focusing on the diagnostic performance for definite PTB. We categorized patients into four groups, by radiographic activity: definitely active, probably active, indeterminate activity, and probably inactive.

**Results:**

Of the 650 patients included, 316 had culture-confirmed PTB; 190 (29.2%), 323 (49.7%), 70 (10.8%), and 67 (10.3%) were classified into the definitely active, probably active, indeterminate activity, and probably inactive groups, respectively. The corresponding observed culture rates for CT radiographic activity were 61.6%, 60.7%, 4.3% and 0%, respectively. When not only culture rates but TB-PCR and histological results were taken into consideration as definite PTB, it showed 66.6%, 67.2%, 14.3%, and 0% of each CT radiographic activity, respectively. Regarding the diagnostic performance for definite PTB, radiographic activity displayed high sensitivity (97.1%, 95% confidence interval (CI), 94.6–98.5) and negative predictive values (92.7%, 95% CI, 86.6–96.2), considered definitely and probably active PTB. Apart from PTB, other etiologies, according to radiographic activity, were predominantly respiratory infections such as bacterial pneumonia and non-tuberculous mycobacterial infection.

**Conclusions:**

Radiographic activity showed good diagnostic performance, and can be used easily in clinical practice. However, clinicians should consider other possibilities, because radiologic images do not confirm microbiological PTB.

## Introduction

Chest computed tomography (CT) plays a significant role in the diagnosis of pulmonary tuberculosis (PTB) [1]. Although chest radiography is used as the first diagnostic method, owing to its rapidity [2], it is limited by poor specificity and reader inconsistency [3–5]. It is important to overcome this disadvantage, therefore. Chest CT can detect early lesions of PTB and distinguish them from other etiologies [1, 6, 7]. Moreover, it can also provide additional information on the mycobacterial activity of TB lesions [8].

Early studies evaluated the pathologic and radiologic correlation using chest CT, and presented the radiological characteristics of PTB, as ‘cavitation’, ‘tree-in-bud’ and ‘consolidation’ [9, 10]. Later, several studies attempted to ascertain the association between each radiographic feature and the smear grade of the sputum or culture yield of *Mycobacterium tuberculosis* (MTB) [11–13]. It is important for clinicians in charge of PTB treatment to anticipate the culture yield of MTB based on CT, to guide proper management. Unfortunately, the results of previously conducted studies, according to each radiographic manifestation, are too complex for diagnostic use in clinical practice, for PTB. Moreover, the culture yield might be underestimated based on the methods used, such as sputum vs. bronchoscopic specimen or culture media. No study so far has addressed these limitations.

Therefore, we evaluated the correlation between the microbiologic yield of MTB and the categorized radiographic grade, on chest CT, via bronchoscopy, in presumptive PTB patients. The categorized grade can be used easily in clinical practice. Bronchoscopy is the most useful and powerful tool for the diagnosis of PTB, and it has discriminative ability [14, 15]. The aim of this study was to (1) determine the relative frequencies of MTB-positive cultures, according to radiographic grades, based on chest CT, in presumptive PTB patients, (2) evaluate the correlation between radiographic activity and PTB (3) identify the other etiology according to radiographic grades.

## Methods

### Study population and design

This retrospective review included patients older than 18 years of age, with suspected PTB, between January 2012 and February 2015. It was performed at the Hallym University Kangdong Sacred Heart Hospital and Inje University Busan Paik Hospital, in the Republic of Korea, a country with an intermediate TB burden and an annual incidence of 86/100,000 persons, in 2014 [16].

As per the enrollment criteria, patients who underwent both chest CT and bronchoscopy for the diagnosis of PTB were included. The exclusion criteria were: 1) patients who had received any anti-TB treatment before bronchoscopy (as it could have affected the mycobacterial culture); 2) patients who were previously treated for PTB, 3) patients with only endobronchial TB, without parenchymal lesions; 4) patients with known diseases associated with parenchymal disease, confused with PTB, such as interstitial lung disease, bronchiectasis and malignancy involving the lung. If the second and fourth criteria were not adhered to, the interpretation of radiographic activity could have been affected.

The study protocol was approved by the institutional review board of each hospital (Kangdong Sacred Heart Hospital and Busan Paik Hospital), and permission was obtained to publish information from patients’ records. The need for informed consent was waived due to the retrospective nature of the study.

### Classification of the radiologic activity of PTB based on chest CT

**Fig 1:**
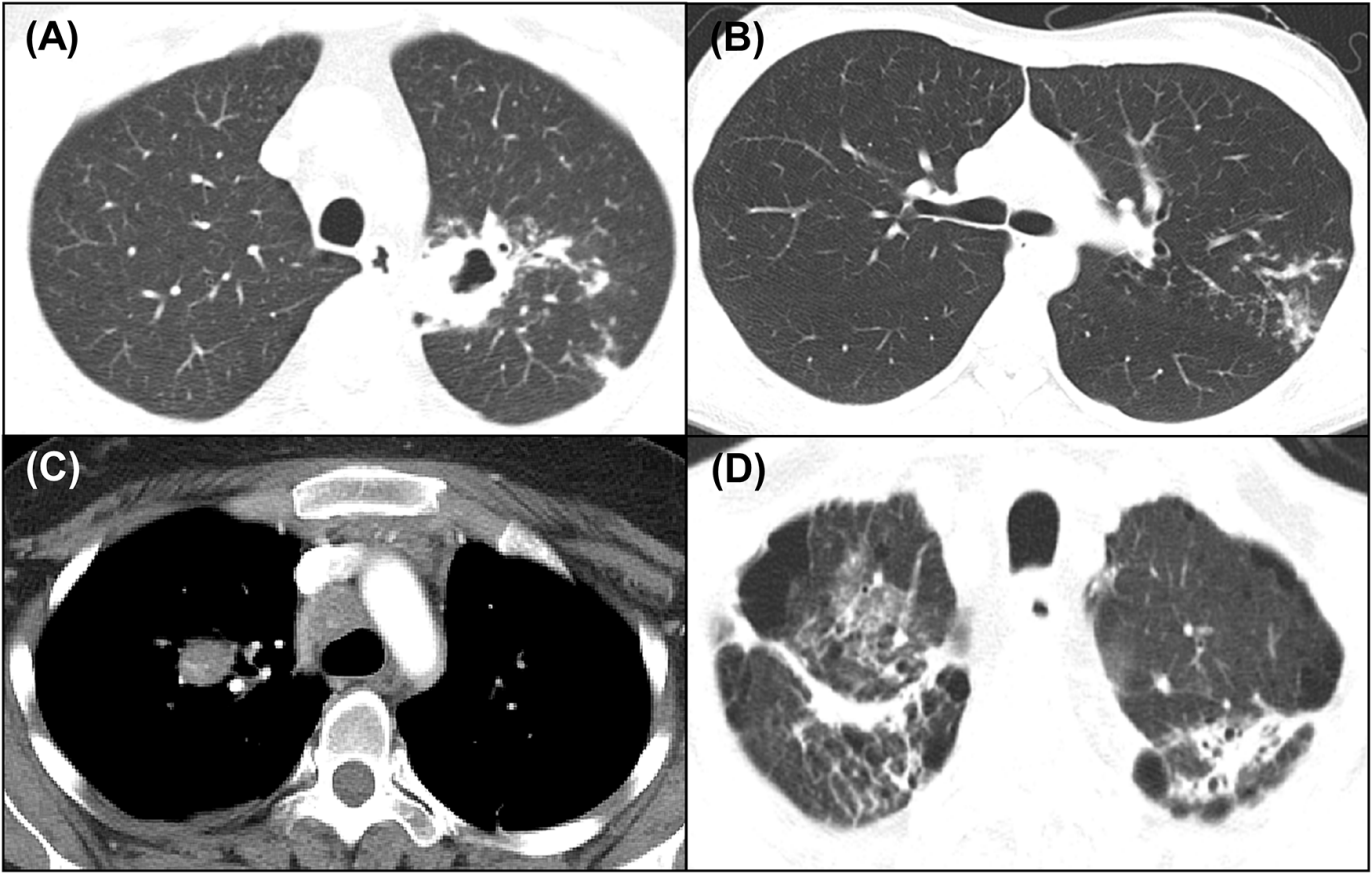
The representative cases according to chest CT classification. (A) definitely-active, with lesions including a cavity (B) probably-active, showing a “tree-in-bud” appearance or multiple non-calcified poorly circumscribed nodules without a cavity (C) indeterminate-activity, with lesions appearing mainly as non-calcified well-circumscribed nodules (D) probably-inactive, with lesions appearing mainly as calcified nodules or fibrotic bands.

Chest CT was reviewed by one radiologist from each institution, and they were all blinded to the microbiological results. Patients with presumptive PTB were categorized into four groups, by radiographic activity, from the highest to lowest grade, based on previously published criteria: definitely active, with lesions including a cavity; probably active, showing a “tree-in-bud” appearance or multiple non-calcified poorly circumscribed nodules without a cavity; indeterminate activity, with lesions appearing mainly as non-calcified well-circumscribed nodules; and probably inactive, with lesions appearing mainly as calcified nodules or fibrotic bands [10, 17–19]. If the lesion of the suggested PTB was in two or more of the above categories, the radiographic activities were determined as being of a higher grade of those. The classification of the radiographic activities is shown in Fig 1.

### Bronchoscopy procedure and microbiological examination

Bronchoscopy was conducted by the faculty staff of the pulmonology division in each hospital. The choice between bronchial washing (BW) and bronchoalveolar lavage (BAL) via bronchoscopy was based on the judgment of individual bronchoscopists. When the multiple lesions of suggested PTB were observed on CT, BW or BAL was conducted on the most severe lesion.

All the bronchoscopic specimens for the Acid-Fast Bacilli (AFB) smears and cultures were processed and pretreated as recommended [20]. The AFB smears were examined after auramine–rhodamine fluorescent staining, and graded on a scale from 0 to 4+.[21] All the specimens were simultaneously cultured on both solid and liquid media using 3% Ogawa medium (Eiken Chemical, Tokyo, Japan) and the mycobacterial growth indicator tube 960 system (Becton Dickinson, Mountain view, CA, USA). TB-PCR was conducted using either the Xpert MTB/RIF assay (Cepheid Inc., Sunnyvale, CA) or AdvanSure TB/NTM RT-PCR kit (LG Life Sciences, Seoul, Korea) [22, 23].

### Definition of PTB and other etiologies

Microbiologically confirmed PTB was defined as the growth of MTB in the culture. Histologically confirmed PTB was defined as the presence of caseating or necrotizing granulomatous inflammation in a tissue sample, with a positive TB-PCR result in that tissue. Genetically confirmed PTB was defined as the detection of positive TB-PCR in the respiratory specimen. Clinically diagnosed PTB was defined as cases in which patients were considered to have PTB, based on symptoms and radiographic findings compatible with PTB, and whose symptoms and radiographic findings improved after anti-TB treatment, even with a negative MTB culture. Of the aforementioned definitions, microbiologically, histologically and genetically confirmed PTB were classified as definite PTB. Cases of clinically diagnosed PTB were classified as probable PTB [24].

Other etiologies were identified as follows. Pulmonary non-tuberculous mycobacterial (PNTM) infection was identified according to the isolation of NTM species and the American Thoracic Society criteria [25]. Pulmonary bacterial infection was identified according to the isolation of compatible pathogenic bacteria in the bronchoscopic specimen and/or a positive urinary antigen test for *Streptococcus pneumoniae* and *Legionella pneumophila* and/or positive PCR for bacterial pathogens such as *Streptococcus pneumoniae*, *Legionella pneumophila*, *Mycoplasma pneumoniae* and *Chlamydia pneumoniae* of the bronchoscopic specimens. Pulmonary viral infection was identified based on the result of a multiplex reverse-transcription PCR assay using a bronchoscopic specimen. Pulmonary fungal infection was identified by the guidelines for each organism [26].

### Statistical analysis

The data are presented as median and IQR (interquartile range) for continuous variables, and as number (percentage) for categorical variables. Data were compared using the Mann–Whitney U test for continuous variables and Pearson’s chi-square test or Fisher’s exact test for categorical variables. To determine the accuracy of each radiographic activity to predict PTB, we estimated the sensitivity, specificity, positive predictive value (PPV), negative predictive value (NPV), positive likelihood ratio (LR+) and negative likelihood ratio (LR-) for each kind of radiographic activity. The Youden index, defined as (sensitivity + specificity) − 1, was calculated for every type of radiographic activity. The cutoff radiographic activity at which the Youden index was the highest was considered the optimal cut-off. All tests were two-sided, and a *P*-value <0.05 was considered significant. Data were analyzed using IBM SPSS Statistics version 24 (IBM Corp., Armonk, NY).

## Results

### Patients’ characteristics

During the study period, 731 patients underwent chest CT and bronchoscopy because of presumed PTB. Of these patients, 81 met the exclusion criteria. Thus, 650 patients were eligible to participate, and they comprised 190 patients (29.2%) classified as the definitely active group, 323 patients (49.7%) as the probably active group, 70 (10.8%) as the indeterminate activity group, and 67 (10.3%) as the probably inactive group. The demographic and descriptive data of the enrolled cases are summarized in Table 1. There were 370 (56.9%) males with a median age of 57.0 (41.0–71.0) years. The enrolled patients had predominantly localized PTB, involving one lobe of the lung parenchyma.

**Table 1:** Demographic and clinical characteristics of 650 patients with presumed PTB.

	No. of patients (%) or median (IQR)
Age, years	57.0 (41.0–71.0)
Male sex, %	370 (56.9)
Comorbidity^a^	
COPD or asthma	28 (4.3)
Thyroid disease	6 (0.9)
Cardiovascular disease	56 (8.6)
Malignancy	21 (3.2)
Hematologic disease	2 (0.3)
Chronic liver disease	25 (3.8)
Rheumatic disease	27 (4.1)
CKD	18 (2.8)
Diabetes	34 (5.2)
Neurologic disease	13 (2.0)
Cerebrovascular disease	19 (2.9)
Immune-suppressive disease	6 (0.9)
HIV infected	2 (0.3)
Bronchoscopic specimen	
BW	430 (66.2)
BAL	220 (33.8)
Radiographic activities based on chest CT	
Definitely active	190 (29,2)
Probably active	323 (49.7)
Indeterminate activity	70 (10.8)
Probably inactive	67 (10.3)
Extent of lung lesion	
Unilobar involvement	378 (57.9)
Multilobar involvement	275 (42.1)

The data are presented as median (interquartile range) or No. (%)

^a^ Cases are duplicated

PTB = pulmonary tuberculosis; IQR = interquartile range; COPD = chronic obstructive lung disease; CKD = chronic kidney disease; HIV = human immunodeficiency virus; BW = bronchial washing; BAL = broncho-alveolar lavage

### Culture yield of MTB according to the radiographic activity on chest CT

**Fig 2:**
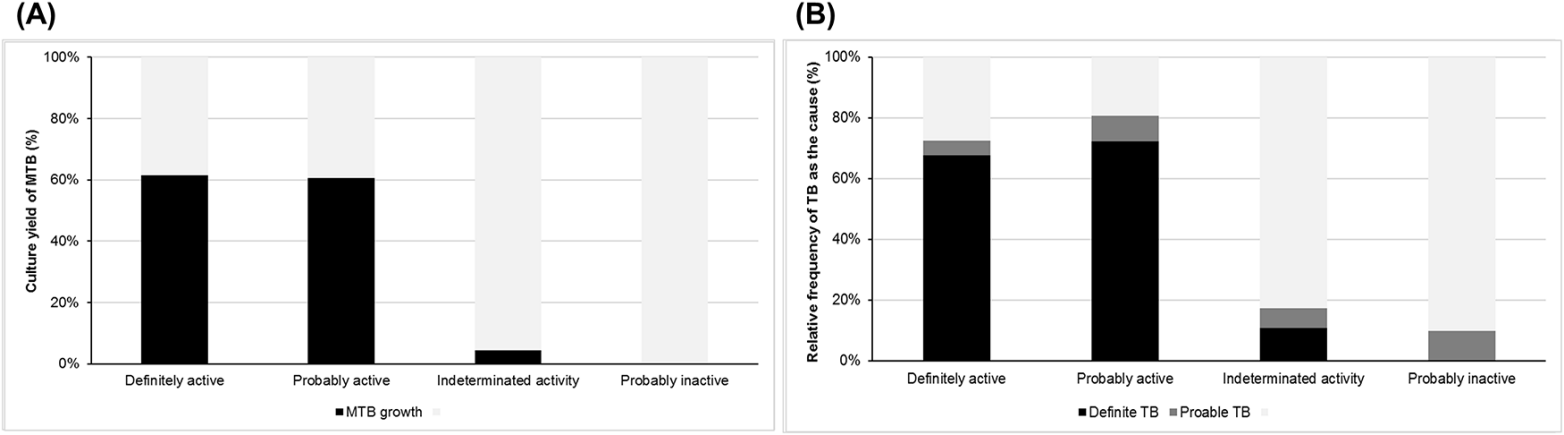
MTB culture yield according to radiographic activities of chest CT (A) and final diagnosis as definite or probable PTB by each radiographic grades (B). MTB = Mycobacterium tuberculosis Definite TB, defined as microbiologically, histologically or genotypically confirmed cases Probable TB, defined as clinically diagnosed case.

The culture yields, according to the radiographic activities, were 61.6%, 60.7%, 4.3% and 0%, respectively (Table 2 and Fig 2). Considering the microbiologically, histologically and genetically confirmed cases, the diagnosis rates of definite PTB according to the radiographic activities were 66.8%, 67.2%, 14.3% and 0%, respectively. When probable PTB was included, the overall rates of PTB diagnosed, according to the radiographic activities, were 71.6%, 75.2%, 22.9% and 9.9%, respectively. In the prediction of PTB, based on the radiographic activities on chest CT, good results were observed for definite or overall PTB. The culture yield of MTB was better in the definitely active group than the probably active group. But, in the prediction of definite and overall PTB, the probably active group had conversely slightly better results than the definitely active group. However, statistical significance was not noted in both.

**Table 2:** Characteristics of study participants according to the radiographic activities on chest CT.

	Definitely active (n = 190)	Probably active (n = 323)	Indeterminate activity (n = 70)	Probably inactive (n = 67)	*P*-value
Age, years	55.0 (42.0–70.0)	55.0 (35.0–69.0)	61.0 (43.0–71.0)	66.0 (53.0–76.0)	<0.001
Male sex, %	130 (68.4)	157 (48.6)	43 (61.4)	40 (59.7)	<0.001
Comorbidity^a^					
COPD and asthma	3 (1.6)	15 (4.6)	3 (4.3)	7 (10.4)	0.022
Thyroid disease	1 (0.5)	5 (1.5)	0	0	0.394
Cardiovascular disease	15 (7.9)	25 (7.7)	6 (8.6)	10 (14.9)	0.281
Malignancy	5 (2.6)	9 (2.8)	3 (4.3)	4 (6.0)	0.516
Hematologic disease	1 (0.5)	1 (0.3)	0	0	0.869
Chronic liver disease	8 (4.2)	10 (3.1)	6 (8.6)	1 (1.5)	0.122
Rheumatic disease	13 (6.8)	8 (2.7)	0	3 (4.5)	0.073
CKD	5 (2.6)	10 (3.1)	1 (1.4)	2 (3.0)	0.892
Diabetes	14 (7.4)	11 (3.4)	4 (5.7)	5 (7.5)	0.201
Neurologic disease	1 (0.5)	9 (2.8)	0	3 (4.5)	0.084
Cerebrovascular disease	7 (3.7)	9 (2.8)	0	3 (4.5)	0.378
Immune-suppressive disease	0	4 (1.2)	0	2 (3.0)	0.117
HIV infected, %	0	1 (0.3)	0	1 (1.5)	0.276
Overall PTB	136 (71.6)	243 (75.2)	16 (22.9)	7 (9.9)	<0.001
Definite PTB	127 (66.8)	217 (67.2)	10 (14.3)	0	<0.001
Microbiologically confirmed PTB	117 (61.6)	196 (60.7)	3 (4.3)	0	
Histologically confirmed PTB	1 (0.5)	3 (0.9)	4 (5.7)	0	
Genetically confirmed PTB	10 (5.3)	18 (5.6)	3 (4.3)	0	
Probable PTB	9 (4.7)	26 (8.0)	6 (8.6)	7 (10.4)	
Extent of lung lesion					0.189
Unilobar involvement	98 (51.6)	193 (59.8)	42 (60.0)	43 (64.2)	
Multilobar involvement	92 (48.4)	130 (40.2)	28 (40.0)	24 (35.8)	

The data are presented as median (interquartile range) or No. (%)

^a^ Cases are duplicated

CT = computed tomography; PTB = pulmonary tuberculosis; IQR = interquartile range; COPD = chronic obstructive lung disease; CKD = chronic kidney disease; HIV = human immunodeficiency virus; BW = bronchial washing; BAL = broncho-alveolar lavage

### Diagnostic performance of the radiographic classification for PTB

The sensitivity, specificity, PPV, and NPV for definite and overall PTB, according to the radiographic activities on CT, are presented in Table 3. In the definitely active and probably active groups, low sensitivity but modest specificity in predicting definite and overall PTB, respectively, were noted. However, when combined, the definitely and probably active groups were considered to suggest active PTB, with high sensitivity and NPV, but low specificity and modest PPV. The optimal cut-off radiographic activities for the prediction of definite and overall PTB, assessed by the Youden index, were over the probably active grade in both.

**Table 3:** Diagnostic performance of the radiographic activities on chest CT in predicting definite and overall PTB in patients with presumed PTB.

Radiographic activity	Sensitivity % (95% CI)	Specificity % (95% CI)	PPV % (95% CI)	NPV % (95% CI)	LR+ % (95% CI)	LP- % (95% CI)	Youden index^a^
Definite PTB							
suggested active PTB	97.1 (94.6–98.5)	42.9 (37.2–48.7)	66.9 (62.7–71.0)	92.7 (86.6–96.2)	2.03 (1.76–2.32)	0.07 (0.04–0.14)	0.40
(combined with definitely and probably active)							
Definitely active	35.8 (30.9–41.1)	78.7 (73.5–83.1)	66.8 (59.6–73.3)	50.6 (59.6–73.3)	2.01 (1.60–2.52)	0.97 (0.88–1.07)	0.15
Probably active	61.2 (55.9–66.3)	64.1 (58.4–69.5)	67.1 (61.7–72.2)	58.1 (52.5–63.4)	2.04 (1.72–2.43)	0.72 (0.63–0.82)	0.25
Indeterminate activity	2.8 (1.4–5.3)	79.7 (74.6–84.1)	14.2 (7.4–25.1)	40.7 (36.7–44.8)	0.167 (0.09–0.29)	1.45 (1.35–1.56)	-0.17
Probably inactive	0	77.3 (72.0–81.9)	0	39.3 (35.3–43.4)	0	1.54 (1.44–1.65)	-0.23
Overall PTB							
Suggested active PTB	94.2 (91.3–96.2)	45.9 (39.6–52.3)	73.8 (69.7–77.5)	83.2 (75.6–88.8)	2.82 (2.41–3.29)	0.20 (0.13–0.29)	0.40
(combined with definitely and probably active)							
Definitely active	33.8 (29.2–38.7)	78.2 (72.4–83.0)	71.5 (64.5–77.7)	42.1 (37.6–46.8)	2.51 (1.97–3.21)	1.37 (1.26–1.49)	0.12
Probably active	60.4 (55.4–65.2)	67.7 (61.4–73.4)	75.2 (70.0–79.7)	51.3 (45.8–56.8)	3.03 (2.48–3.71)	0.94 (0.84–1.06)	0.28
Indeterminate activity	3.9 (2.3–6.5)	78.2 (72.4–83.0)	22.8 (14.0–34.7)	33.5 (29.6–37.5)	0.29 (0.18–0.46)	1.98 (1.86–2.12)	-0.18
Probably inactive	1.7 (0.7–3.7)	75.8 (69.8–80.9)	10.4 (4.6–20.9)	32.3 (28.5–36.2)	0.11 (5.7–23.6)	2.10 (1.97–2.23)	-0.22

^a^Youden index = ((sensitivity + specificity) − 1).

CT = computed tomography; PTB = pulmonary tuberculosis; CI = confidence interval; PPV = positive predictive value; NPV = negative predictive value; LR+ = positive likelihood ratio; LR− = negative likelihood ratio.

### Other etiologies according to the radiographic activities on chest CT

Table 4 shows the isolated or detected microorganisms, other than MTB. When organisms other were classified according to the radiographic activities, no remarkable differences were observed. Table 5 shows the final diagnosis of the enrolled cases, according to the radiographic activities. In almost all the cases except PTB, bacterial pneumonia as the cause of parenchymal lung lesion was the most prevalent, followed by PNTM. We further analyzed the distribution of the microbial etiologies in the cases finally diagnosed as bacterial pneumonia (Table 6), but no statistical differences was not noted.

**Table 4:** Microbiological etiology other than PTB according to the radiographic activities on chest CT.

	Definitely active (n = 190)	Probably active (n = 323)	Indeterminate activity (n = 70)	Probably inactive (n = 67)	*P*-value
NTM	16 (8.4)	22 (6.8)	9 (12.9)	4 (6.0)	0.348
*Mycobacterium absscessus*	0	2	0	0	
*Mycobacterium masilliense*	0	2	0	0	
*Mycobacterium avium*	4	3	3	1	
*Mycobacterium intracellulare*	9	15	4	2	
*Mycobacterium kansasii*	3	0	0	1	
*Mycobacterium smegmatis*	0	0	2	0	
Bacteria					
*Streptococcus pneumoniae*	4 (2.1)	3 (0.9)	1 (1.4)	0	0.513
Other streptococcus spp.	50 (26.3)	98 (30.3)	17 (24.3)	21 (31.3)	0.604
*Staphylococcus aureus*	0	7 (2.2)	1 (1.4)	0	0.135
*Pseudomonas* spp.	2 (1.1)	3 (0.9)	3 (4.3)	0	0.090
*Stenotrophomonas maltophilia*	1 (0.5)	0	0	1 (1.5)	0.206
*Mycoplasma pneumoniae*	0	1 (0.3)	0	0	0.489
*Klebsiella* spp.	10 (5.3)	7 (2.2)	3 (4.3)	3 (4.5)	0.293
*Haemophilus influenzae*	0	3 (0.9)	0	0	0.384
*Enterobacter* spp.	1 (0.5)	1 (0.3)	0	0	0.869
*Acinetobacter* spp.	2 (1.1)	0	0	0	0.183
Others	10 (5.3)	28 (8.7)	5 (7.1)	8 (11.9)	0.301
Fungus					
*Candida* spp.	8 (4.2)	6 (1.9)	0	1 (1.5)	0.153
Virus	3 (1.6)	3 (0.9)	1 (1.4)	2 (3.0)	0.615
Cytomegalovirus	0	1	0	1	
Corona virus	1	0	0	0	
HSV type B	0	1	0	0	
Parainfluenza	1	0	0	0	
RSV-A	0	0	1	0	
Rhino virus	1	1	0	1	

CT = computed tomography; NTM = non-tuberculous mycobacteria; spp = species; HSV = herpes simplex virus; RSV = Respiratory syncytial virus

**Table 5:** Final diagnosis of 650 patients with presumed PTB.

	Definitely active (n = 190)	Probably active (n = 323)	Indeterminate activity (n = 70)	Probably inactive (n = 67)
PTB	136 (71.6)	243 (75.2)	16 (22.9)	7 (10.4)
PNTM	15 (7.9)	21 (6.5)	9 (12.9)	3 (4.5)
Bacterial pneumonia	28 (14.7)	43 (13.3)	37 (52.9)	28 (41.8)
LCA	6 (3.2)	4 (1.2)	1 (1.4)	0
CCPA	1 (0.5)	0	0	0
PJP	0	1 (0.3)	0	0
Sarcoidosis	0	1 (0.3)	2 (2.9)	0
Viral pneumonia	0	2 (0.6)	0	0
Unidentified	4 (2.1)	8 (2.5)	5 (7.1)	29 (43.3)

PTB = pulmonary tuberculosis; PNTM = pulmonary non-tuberculous mycobacteria; LCA = lung cancer, CCPA = chronic cavitary pulmonary aspergillosis; PJP = pneumocystis jirovecii pneumonia

**Table 6:** Microbial etiology of finally diagnosed bacterial pneumonia according to radiographic activity.

	Definitely active (n = 31)	Probably active (n = 44)	Indeterminate activity (n = 37)	Probably inactive (n = 29)	*P*-value
Bacterial Pneumonia					
*Streptococcus pneumoniae*	4 (10.5)	1 (2.6)	1 (2.0)	0	0.712
Other streptococcus spp.	5 (17.2)	6 (15.4)	10 (20.0)	3 (10.3)	0.730
*Staphylococcus aureus*	0	1 (2.6)	3 (6.0)	0	0.301
Pseudomonas spp.	1 (3.4)	3 (7.7)	1 (2.0)	0	0.320
*Stenotrophomonas maltophilia*	0	0	0	1 (3.4)	0.251
*Mycoplasma pneumoniae*	0	0	0	0	NA
Klebsiella spp.	5 (17.2)	2 (5.1)	2 (4.0)	3 (10.3)	0.171
*Haemophilus influenzae*	0	0	0	0	NA
Enterobacter spp.	0	0	0	0	NA
Acinetobacter spp.	0	0	0	0	NA
Others	1 (3.4)	3 (7.7)	6 (12.0)	3 (10.3)	0.613

Cases with no growth of bacteria in the lower respiratory specimen or other urologic, serologic tests are not presented in this table

## Discussion

The study evaluated the correlation between microbiological yield and radiographic activity on chest CT, in suspected PTB patients. In this real-world study, the culture rates of each radiographic activity were 61.6%, 60.7%, 4.3% and 0%, respectively. However, with regards to the diagnostic performance of definite and overall PTB, the radiographic activities showed high sensitivity and NPV when the definitely and probably active grades were considered as active PTB. These findings suggest that radiographic activity can be used for the diagnosis of PTB, and in the initial guidance of anti-TB therapy, for presumptive PTB, in clinical practice. This is the first study to evaluate the correlation of microbiologic yield with radiographic activities on chest CT, in suspected PTB cases, in real-world settings.

PTB has a long history of challenging physicians more than any other infectious lung disease, due to the great morbidity and mortality associated with it. Moreover, in addition to the outcome, the diagnosis of PTB is also challenging because, although microbial identification of MTB is the gold standard in the diagnosis of PTB, it is frequently not properly performed and is relatively time-consuming, despite recent advances in the culture methods [27]. So, chest radiography or CT plays a critical role in the diagnosis of PTB and initial guidance of anti-TB therapy for presumptive PTB patients [3, 12]. CT is more advantageous than radiography in several aspects and is widely used even now [19, 28–30]. However, little has been reported on the correlation of microbiological yield with radiographic activities on CT, in suspected PTB cases. Moreover, the shadow of the lung lesion in CT does not confirm microbiological PTB [30–34].

Researchers have, in the past, tried to define the radiographic characteristics and activities of PTB for its early detection and to prevent its transmission, as it has threatening outcomes. In many previously conducted studies, several radiologic features, such as cavity, centrilobular nodules, tree-in-bud, air-space consolidation, etc., and locations such as the upper lobe or superior segment of the lower lobes, were suggestive of active PTB [8, 10, 12, 17, 18, 29–33, 35]. Moreover, some research studies classified and provided the radiographic criteria for PTB activity based on chest CT [10, 14, 17, 18].

However, radiographic findings alone, despite CT scans proving valuable in clinical practice, cannot provide a definitive diagnosis of PTB, because radiologic similarity has been observed in other disease entities too [30–34]. Therefore, the microbiological confirmation of PTB should be done carefully. Recently, liquid culture systems have been developed and are widely used to hasten and augment the yield of the MTB culture; the World Health Organization also recommends the widespread adoption of liquid culture [35]. However, many previously conducted studies focused on the identification of the radiologic characteristics and activities based on histological correlation, or the microbiological confirmation mostly through sputum [9, 10, 17, 18, 30, 33, 35]. Several studies have focused on each radiologic pattern, such as tree-in-bud or cavity, and its cause [32, 34, 36, 37], but there are no data with regards to the overall causes of presumptive PTB, based on chest CT, according to the grade of the radiographic activities. Although it is important to identify the radiologic characteristics of definite PTB, it is conversely, in clinical practice, more important and useful to identify the relative frequency of definite PTB in presumptive PTB patients, based on a chest CT scan.

For these reasons, we conducted this study using a concept borrowed from the radiographic criteria for PTB activities based on chest CT [10, 14, 17, 18]. We aimed to estimate the relative incidence of microbiologically confirmed PTB, by bronchoscopy, which is regarded as the most powerful diagnostic method for PTB, according to previously published criteria of radiographic activity based on a chest CT scan. In this study, we identified a significant correlation between radiographic activity, according to the morphological characteristics of chest CT, and the microbiological confirmation of PTB. This information will prove useful to clinicians for the diagnosis of PTB and in the initial guidance of anti-TB therapy for presumptive PTB patients.

There are several potential limitations to our study that should be acknowledged. First, given its retrospective nature, selection bias may have influenced our findings. In this study, we only enrolled patients who underwent bronchoscopy because, through it, other etiologies can be differentiated from PTB. Moreover, the proportion of definite PTB cases corresponding to each radiographic activity could have been underestimated because the enrolled patients predominantly had localized disease (57.9%), and were determined as requiring bronchoscopy for diagnosis. However, this inclusion could have decreased the proportion of undiagnosed cases, unlike previous other studies [34, 37]. Second, the CT image protocol was not the same in all the enrolled patients. This may have affected the morphological characteristics required for some subtle findings. Third, we conducted this study in South Korea, an intermediate TB burden county, so this result should be interpreted with infectious etiology of each lesion. For example, the most prevalent cause of tree-in-bud appearance on chest CT is PNTM infection in US study, while PTB and pneumonia due to *Streptococcus pneumonia and Haemophilus* species in Israel study [34, 37]. Moreover, cavitary lesion of lung is also diagnosed other than PTB [32, 36]. Thus, this result of study should be applied in other clinical setting with caution.

In conclusion, the radiographic activities, based on chest CT, showed a modest correlation with the microbiological yield of MTB, and in combination with definitely and probably active lesions showed a good performance for the diagnosis of PTB. The results of this study suggest that the radiographic activity, based on chest CT can be used easily in clinical practice. However, clinicians should consider possibilities other than PTB, because radiologic images do not confirm microbiological PTB.

## Supporting information

S1 File Raw data. (XLS)
